# *Crosspteryx fibrifuga* leaf extract enhances host resistance to *Trypanosoma congolense* infection in mice by regulating host immune response and disrupting the activity of parasite superoxide dismutase enzyme

**DOI:** 10.3389/fmicb.2023.1275365

**Published:** 2023-10-25

**Authors:** Nnamdi Ikeogu, Folayemi Olayinka-Adefemi, Chidalu Edechi, Chukwunonso Onyilagha, Ping Jia, Aaron Marshall, Julius Ode, Jude Uzonna

**Affiliations:** ^1^Department of Immunology, University of Manitoba, Winnipeg, MB, Canada; ^2^Department of Pathology, University of Manitoba, Winnipeg, MB, Canada; ^3^National Centre for Foreign Animal Disease, Canadian Food Inspection Agency, Winnipeg, MB, Canada; ^4^Department of Veterinary Pharmacology and Toxicology, University of Abuja, Abuja, Nigeria

**Keywords:** *Crosspteryx fibrifuga*, *Trypanosoma. Congolense*, superoxide dismutase, interferon-gamma, host response

## Abstract

African trypanosomiasis, a neglected tropical disease, is caused by diverse species of the protozoan parasite belonging to the genus *Trypanosoma*. Although anti-trypanosomal medications exist, the increase in drug resistance and persistent antigenic variation has necessitated the development of newer and more efficacious therapeutic agents which are selectively toxic to the parasite. In this study, we assessed the trypanocidal efficacy of *Crosspteryx fibrifuga* leaf extract (*C.f/L*-extract) *in vitro*. Following treatment of *T. congolense* parasites with *C.f/L*-extract, we observed a significant decrease in parasite number and an elevation in the expression of the apoptotic markers, Annexin V and 7-Aminoactinomycin D (7AAD). Interestingly, at the same concentration (50 μg/mL), *C.f/L*-extract was not cytotoxic to murine whole splenocytes. We also observed a significant increase in pro-inflammatory cytokines and nitric oxide secretion by bone marrow derived macrophages following treatment with *C.f/L-extract* (10 μg/mL and 50 μg/mL) compared to PBS treated controls, suggesting that the extract possesses an immune regulatory effect. Treatment of *T. congolense* infected mice with *C.f/L*-extract led to significant decrease in parasite numbers and a modest increase in mouse survival compared to PBS treated controls. In addition, there was a significant increase in CD4^+^IFN-γ^+^ T cells and a decrease in CD4^+^IL-10^+^ T cells in the spleens of *T. congolense* infected mice treated with *C.f/L*-extract. Interestingly, *C.f/L*-extract treatment decreased the activity of superoxide dismutase (an enzyme that protects unicellular organisms from oxidative stress) in *T. congolense* parasites but not in splenocytes. Collectively, our study has identified C.f/L-extract as a potential anti-trypanosomal agent that warrant further investigation and possibly explored as a treatment option for *T. congolense* infection.

## Graphical Abstract

Following infection with *T. congolense* parasites in mice, *C.f/L-extract* administration increased production of IL-12p40, IL-6, TNF-α, and NO in macrophages. CD4^+^T cells from infected mice treated with *C.f/L-extract* contained increased frequency of CD4^+^IFN-γ^+^ and decreased CD4^+^IL-10^+^. *C.f/L-extract* treatment caused a significant reduction in parasite superoxide dismutase (SOD) activity, leading to increased parasite death, decreased parasitemia and consequently increased host survival.
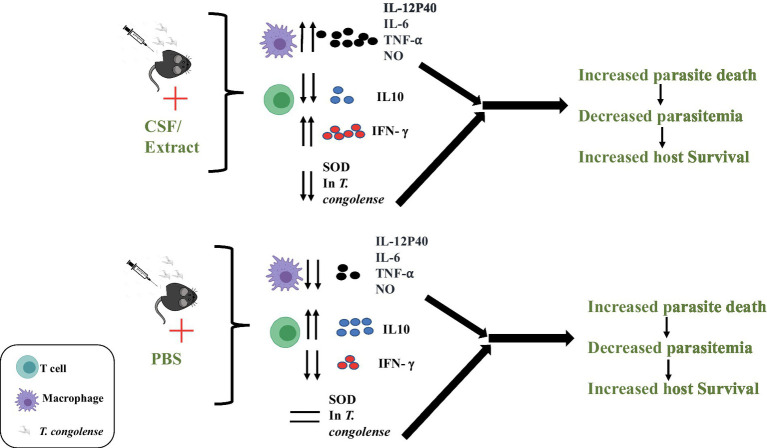

## Introduction

In endemic regions, African trypanosomiasis is a disease of health and economic importance in humans and animals (CDC, 2019). It is caused by blood protozoan parasites of the genus *Trypanosoma*, and transmitted by the insect vector, tsetse fly (*Glossina spp*) (WHO, 2019). In humans, African trypanosomiasis is caused by *Trypanosoma brucei rhodesiense* and *Trypanosoma brucei gambiense*. In addition to the classic sleeping sickness disease, African trypanosomiasis results in severe morbid symptoms such as fever, lethargy, nervous disorders and can be highly fatal if untreated (Stich et al., 2002; Mwanakasale et al., 2014). *Trypanosoma congolense* is the specie of concern in pet and food animals because billions of dollars are lost per year due to the disease caused by this species in animals (Holt et al., 2016).

There are currently no vaccine against the disease and effective and safe treatment options are highly limited (Babokhov et al., 2013). In humans, the available treatments include use of Pentamidine and Suramin, which are often given in the early stages of the disease. During the later (chronic) stages, Melarsoprol and Eflornithine are the drugs of choice (Babokhov et al., 2013). The drawbacks with the use of these existing drugs include drug toxicity, tissue damage, laborious administration of the drug and the technical expertise required for administration (Babokhov et al., 2013; CDC, 2019; WHO, 2019). The increase in parasite resistance to some of these available drugs is another setback with current anti-trypanosomal therapy. For example, increase in occurrence of *T. congolense* strains that are resistant to the broad-spectrum anti-protozoan drug diminazene aceturate (Berenil) have been reported in some natural infections in cattle (Mbwambo et al., 1988). Besides, Berenil is also highly toxic in dogs (Mbwambo et al., 1988). Hence it is necessary to develop novel and safe therapeutic options against the disease.

*Crosspteryx fibrifuga* is a deciduous flowering tree found in East, West, and Central Africa (Adeola et al., 2011). Extracts from seeds, bark, roots and leaves of *C. fibrifuga* are known to have medicinal properties that have been adopted in traditional medicine as anti-pyretic, analgesic and anti-inflammatory agents (Salawu et al., 2013). The methanolic extract of the root bark of *Crosspteryx fibrifuga* was reported to show antimicrobial properties (Adeola et al., 2011) and anti-plasmodial effects (Salawu et al., 2013). However, it was also reported to lack anti-trypanosomal effect in *T. congolense* infected rats (Yusuf et al., 2006).

Here we report that *C. fibrifuga* leaf extracts (C.f/L-extracts) was selectively trypanocidal to *T. congolense* parasites as evident by a significant decrease in parasite number in *in-vitro* cultures and increased expression of apoptotic markers in the parasites following exposure to *C.f/L*-extracts. Furthermore, *C.f/L*-extracts disrupts the parasite-dependent enzyme, superoxide dismutase (SOD) which is known to protect the parasites from free radical attacks (Prathalingham et al., 2007). Similarly, the administration of *C.f/L*-extracts to *T. congolense* infected mice led to a marked decrease in parasitaemia and improved mice survival when compared to untreated controls. This was associated with enhanced CD4^+^IFN-γ^+^ T cell response in the spleens and increased levels of proinflammatory cytokines in the plasma.

Altogether, our findings show that the methanol leaf extract of *C. fibrifuga* selectively destroys *T. congolense* with minimal toxicity to host cells thereby providing a possible novel therapeutic option for *T. congolense* infection in endemic areas.

## Materials and methods

### Ethics statement

All studies involving laboratory animals were conducted according to the regulation and guidelines of the Canadian Council on Animal Care and approved by the University of Manitoba Animal Care Committee (Protocol number 18-013/1/2/3).

### Mice

Female CD1 (outbred Swiss white mice) and Balb/c mice weighing an average of 20 grams (g) (6–8 weeks old) were obtained from the University of Manitoba Central Animal Care Services (CACS) and used for all animal experiments. The mice were housed, maintained and used according to the recommendations of the Canadian Council on Animal Care.

### Parasite isolation, infection, and estimation

The Trans Mara strain clone TC13 (TC13) of *T. congolense* whose origin has previously been documented (Tabel, 1982) was used for *in vitro* and *in vivo* experiments. In obtaining parasites, CD1 mice were immunosuppressed intraperitoneally (i.p) using cyclophosphamide (Cytoxan; 200 mg/kg) and infected with TC13 stabilates i.p. after 48 h (h) (Tabel, 1982). Blood containing parasites was obtained via cardiac puncture after 72 h of infection, and the parasites were isolated by anion exchange chromatography with the use of diethylamino ethyl (DEAE) cellulose. Parasites were washed twice with Tris-saline glucose (TSG) and resuspended in TSG containing10% Fetal Bovine Serum (TSG-FBS). For *in vivo* studies, mice were infected with 1×10^3^ parasites in TSG-FBS intraperitoneally. Parasite burden in infected mice was estimated from a drop of blood via the tail vein as previously reported (Tabel, 1982).

### Generation of single cells (splenocytes) from whole spleens

Single cell suspensions from whole spleens were generated as previously described (Liu and Uzonna, 2010). Briefly, mice were sacrificed by euthanasia with isoflurane according to approved protocols. The spleens were harvested from euthanized mice and made into single cell suspensions by passing them through sterile cell strainers (Thermo Fisher, Canada). The cell suspensions were pelleted at 1200 rpm for 5 min, and red blood cells were lysed using RBC lysis buffer (Thermo Fisher) according to the manufacturer’s suggested protocol. Next, the cells were pelleted as above and resuspended in complete DMEM (DMEM supplemented with 10% FBS, 100 U/mL penicillin, 2 mmol L-glutamine and 100 μg/mL streptomycin) at the desired concentrations depending on the particular experiment.

### XTT assay

XTT cell viability assay kit (CyQUANT) from Thermofisher (Toronto, Canada) was used to measure the viability of splenocytes following exposure to *C.f/L*-extract. Briefly, 5 × 10^6^ splenocytes were cultured in complete RPMI medium (RPMI 1640 medium supplemented with 10% heat-inactivated FBS, and 100 U/mL penicillin/streptomycin, 5 × 10^−5^ 2-ME, and 2 mM L-glutamine) and treated with PBS, 10 μg/mL, 50 μg/mL, and 100 μg/mL of *C.f/L*-extract for 24 h at 37°C. After 24 h, the viability of cells was assessed using CyQUANT kit reagents according to the manufacturer’s instructions. Absorbance was measured at wavelengths of 450–660 nm using spectra Max 190 from Molecular Devices (San Jose, United States). The absorbance values were normalized, and cell viability were represented in percentages.

### Cell counting by trypan blue exclusion

Five million (5 × 10^6^) splenocytes were cultured in complete RPMI media and treated with PBS, 10 μg/mL, 50 μg/mL and 100 μg/mL of *C.f/L*-extract for 24 h at 37°C. After 24 h, cells were mixed with trypan blue (1:1) and counted using a TC-19 cell counter (Bio-Rad Laboratories, Canada).

### Bone marrow derived macrophage isolation

Bone marrow-derived macrophages (BMDMs) were obtained from bone marrow cells as previously described (Ikeogu et al., 2020). Briefly, bone marrow cells (2 × 10^5^/mL) were plated in petri dishes in complete medium RPMI 1640 medium supplemented with 30% L929 cell culture supernatant at 37°C. After 8 days, the percentage of F4/80^+^ cells (~90%) was determined by flow cytometry.

### Plant identification and collection

The leaves of *C. febrifuga* were collected in September 2017 from Orba community in Nsukka Local Government Council of Enugu State, Nigeria (6.8429°N, 7.3733°E). The plant was identified by Mr. Ozioko Alfred (Taxonomist) in the Department of Botany, Faculty of Biological Sciences, University of Nigeria, Nsukka. The specimen is currently stored and deposited at the herbarium unit of the department for reference.

### Drying, extraction of plant material, and dissolution of *Crosspteryx febrifuga* leaf extract

The *C. febrifuga* leaf was air dried and continuously weighed until a constant mass was achieved. The dried leaves were pulverized and stored in an airtight container until ready for use. Extraction was performed by mixing 200 g of *C. febrifuga* leaf powder in 80% methanol at a temperature of 64°C for 8 h using soxhlet extractor. The extracts were concentrated at 40°C in a rotary evaporator and later stored at −20°C. When needed for experiments, extract was diluted in PBS to the desired concentrations.

### *In vivo* assessment of anti-trypanosomal effect of *Crosspteryx febrifuga* leaf extract

Mice were infected with 1 × 10^3^ *T. congolense* intraperitoneally (i.p.). At 3 days post infection, mice were weighed (~20 g) and placed into 3 groups of 10 mice per group and *C. febrifuga* leaf extract was administered i.p. for 5 days at a dose of 0.5 μg/g and 2.5 μg/g to group 1 and group 2 mice, respectively. Mice in group 3 were the control group and received PBS only for the same duration as group 1 and group 2 mice. At selected time points, parasitemia was estimated as previously described (Tabel, 1982).

### Intracellular cytokine detection by flow cytometry

At the indicated time point (8 days after infection) mice were sacrificed, and single-cell suspensions (splenocytes) were obtained from their spleens as previously described (Liu and Uzonna, 2010). The cells were stimulated with PMA (20 ng/mL), ionomycin (1 μM), and Brefeldin A (10 μg/mL) (all from Sigma) for 4 h, stained with fluorochrome-conjugated antibodies against CD3 and CD4 molecules and fixed for 15 min with 0.5 mL 2% paraformaldehyde (Sigma) as done previously (Ikeogu et al., 2021). For the detection of intracellular cytokines, the cells were permeabilized with 0.1% saponin (Sigma) in FACS buffer for 15 min on ice, washed with FACS buffer and stained for intracellular cytokine (IL-10, IFN-γ, and TNF-α,) with fluorochrome-conjugated antibodies (eBioscience; each at 0.5 μL per tube) for 30 min on ice. Afterwards, the cells were washed with FACS buffer containing 0.1% saponin and assessed for intracellular cytokine using FACS Canto II flow cytometer (BD Bioscience, Mississauga, ON, Canada) and analyzed with FlowJo software (TreeStar, Ashland OR).

### *In vitro* toxicity and trypanocidal assay for *Crosspteryx fibrifuga* leaf extract, direct staining, and flow cytometry

Single cell suspensions of spleen cells from mice were made and RBC contaminants were lysed with ACK lysis buffer. The cells were washed subsequently with PBS and afterwards resuspended in complete tissue culture media at a final concentration of 2 × 10^6^/mL in (DMEM supplemented with 10% FBS 100 U/mL penicillin, 2 mmol L-glutamine, 100 μg/mL streptomycin) and treated with 10 μg/mL, 50 μg/mL, and 100 μg/mL of *Crosspteryx fibrifuga* leaf extract in tissue culture plates for 24 h. Similarly, live parasites isolated from the blood of *T. congolense* infected CD1 mice were resuspended at a concentration of 10 × 10^6^/mL in TSG + 10% FBS solution and treated with 10 μg/mL and 50 μg/mL *Crosspteryx fibrifuga* leaf extract in tissue culture plates for 24 h.

Splenic cells and parasite viability were assessed via Annexin V and 7-AAD staining (eBioscience), according to the manufacturer’s instruction and were analysed by flow cytometry using a BD FACS Canto II cytometer (BD Bioscience, San Diego, United States). Further analyses were conducted on the FlowJo software (BD Bioscience).

### Cytokines and nitrite determination

Bone marrow derived cells (BMDMs, 2 × 10^5^/mL) were treated with PBS, 10 μg/mL or 50 μg/mL *Crosspteryx fibrifuga* leaf extract. After 24 h, the amount of IL-6, TNF-α, and IL-12p40 in the various culture supernatant were analysed via ELISAs using antibody pairs from BD Biosciences following the manufacturer’s instructions. In other experiments, the quantities of IL-10, IFN-γ, and TNF-α were also assessed via ELISA (at sensitivities of 10, 2 and 5 pg./mL) from the plasma of mice treated with PBS, 0.5 μg/g or 2.5 μg/g of *Crosspteryx fibrifuga* leaf extract. The sensitivities of the ELISA were 2, 10, 15, and 31 pg./mL for IFN-γ, IL-10, IL-6/IL-12, and TNF-α, respectively. Furthermore, 2 × 10^5^/mL BMDMs were treated with PBS, 10 μg/mL or 50 μg/mL *Crosspteryx fibrifuga* leaf extract for 24 h, and nitrite concentration in the supernatant fluid was assayed via the standard Griess reaction (Uno et al., 2010).

### Superoxide dismutase and catalase activity

20 × 10^6^ *T. congolense* trypomastigotes were cultured in TSG containing 10% Fetal Bovine Serum (TSG-FBS) for 24 h in the presence of 10 μg/mL and 50 μg/mL of *Crosspteryx fibrifuga* leaf extract and PBS was used as control. Afterwards, the parasites were centrifuged at 1000 g for 10 min at 4°C and the pellets recovered. The pellets were suspended in 3 mL of TSG buffer and were sonically disintegrated in three cycles of 30 s each at 60 W. The resultant homogenate was centrifuged at 1500 g for 5 min at 4°C, and the pellet was washed thrice in ice-cold TSG buffer. The washed pellets (in TSG Buffer) were further centrifuged at 2500 g for 10 min at 4°C and the resultant supernatant was collected. (13)Superoxide dismutase (SOD) and Catalase (CAT) activities were determined in the supernatants using the kit from Abcam (Toronto, Canada) according to the manufacturer’s instruction.

### Statistical analysis

All data are presented as mean and standard error of mean (SEM). Using GraphPad prism program (GraphPad Software Inc., CA, United States), ANOVA and Student’s *t*-tests were used to compare means and SEM between two groups. Error bars indicate ± SEM and differences considered significant at *p* < 0.05.

## Results

### *Crosspteryx Fibrifuga* leaf extract (*C.F/L-extract*) is non-cytotoxic to host cells at low concentration

**Figure 1:**
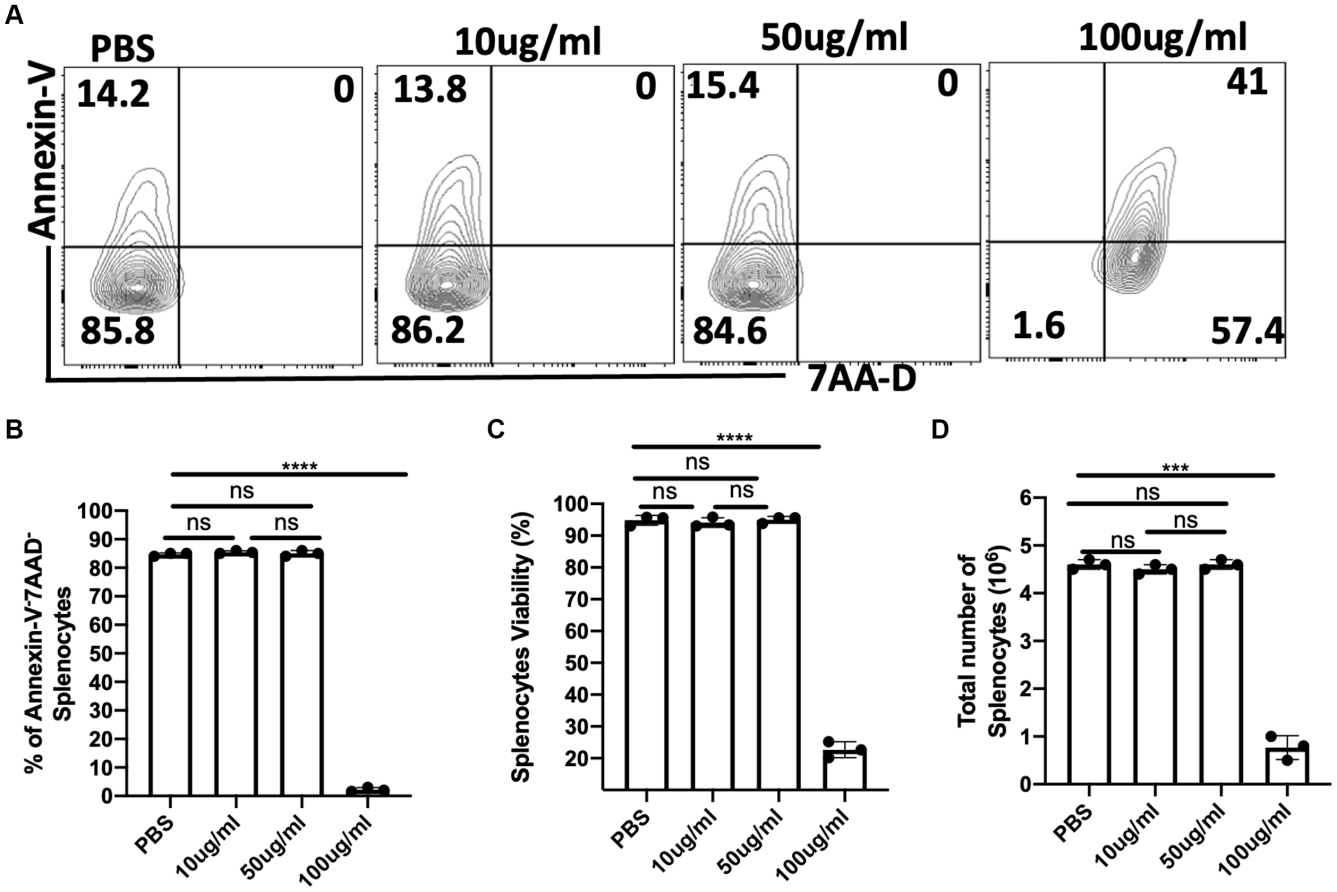
Lower concentrations of *C. fibrifuga* leaf extract (*C.f/L-extract*) is non-cytotoxic to host cells. Splenocytes (5 × 10^6^/mL) were cultured in 24-well tissue culture plates in RPMI medium supplemented with 10% heat-inactivated FBS (complete cell culture medium) in the presence or absence of varying concentrations of *C.f/L*-extract as indicated. After 24 h, the cells were analysed for the expression of Annexin-V and 7AAD by flow cytometry **(A)** and the frequency of live cells (Annexin-V^−^7AAD^−^) were presented graphically **(B)**. In addition, the viability of the cells were assessed by the XTT cell viability assay **(C)**. Viable cells (trypan blue negative cells) were counted with a haemocytometer and the numbers are presented graphically **(D)**. The results presented are representative of 3 different experiments with similar outcome: ns, not significant.

Selective toxicity is a hallmark of an ideal antimicrobial compound (Purssell, 2019). To assess the toxicity of *C.f/L*-extract on host cells, we cultured spleen cells from Balb/c mice in complete media with different concentrations (10 μg/mL, 50 μg/mL, and 100 μg/mL) of the extract and after 24 h, assessed cell survival by flow cytometry after staining for Annexin-V and 7AAD. The viability of the cells was also estimated using the XTT viability assay and cell counts on the hemocytometer. We observed that similar numbers of Annexin-V^+^ cells in the controls (PBS) and groups treated with lower concentrations (10 μg/mL and 50 μg/mL) of the extract. Furthermore, at 10 μg/mL and 50 μg/mL of *C.f/L*-extract there was no double positive (Annexin-V^+^7AAD^+^) cell populations (Figures 1A,B). Additionally, assessment of live cells using the XTT assay (Figure 1C) and hemocytometer counts (Figure 1D) further corroborated the absence of a cytotoxic effect with *C.f/L*-extract at lower concentrations. However, at a higher concentration (100 μg/mL), *C.f/L*-extract was toxic to host cells as there was significant reduction in the numbers of live cells (Figures 1A–D). These results strongly suggest that at low concentrations (10 μg/mL and 50 μg/mL), *C.f/L*-extract is not cytotoxic to host cell. However, at higher concentration (100 μg/mL), it may become toxic to host cells. Therefore, subsequent studies were carried out using low concentrations of the extract.

### C.F/L-extract decreases parasitemia and promotes survivability in *Trypanosoma Congolense* infected mice

**Figure 2:**
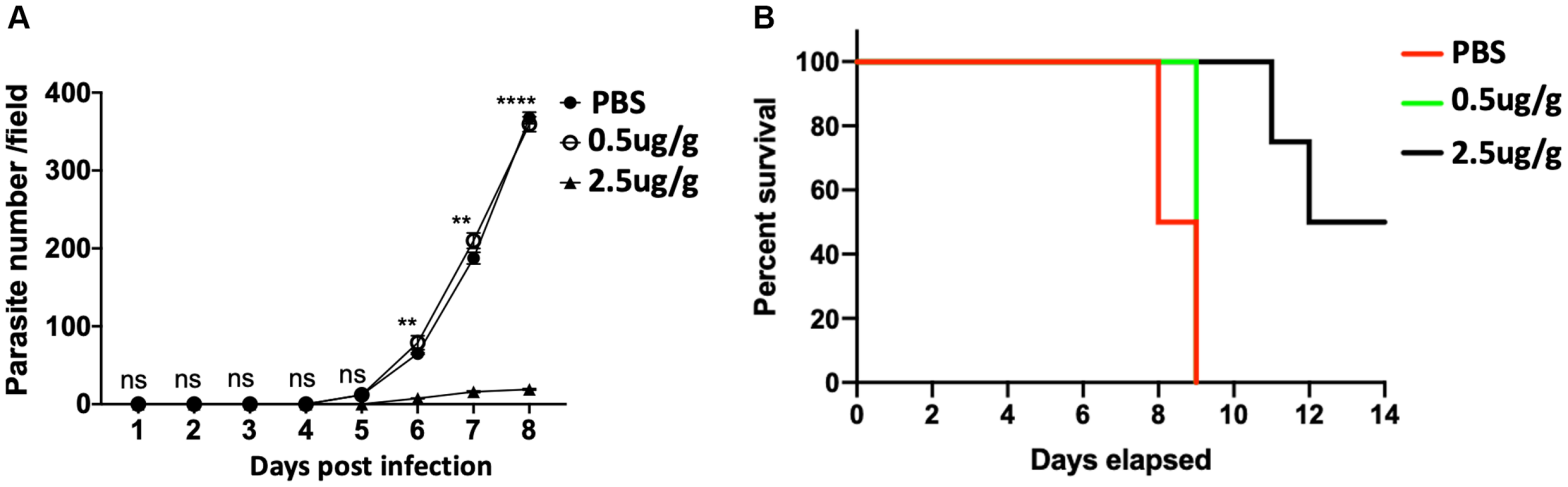
Decreased parasite number and increased survival of *T. congolense* infected mice treated with *Crosspteryx fibrifuga* leaf extract Thirty (Kumar and Tarleton, 2001) Balb/c mice were infected with 1 × 10^3^ *T. congolense* (clone TC13) intraperitoneally (i.p) and treated daily with either PBS or different doses (10 mice per group as indicated) of *C.f/L*-extract starting from 3 days after infection. Parasitemia was monitored daily for 8 days by counting the number of parasites/fields on a wet blood mount **(A)**. Percent survival of the infected animals was graphically represented **(B)**. Results presented are representative of 2 different experiments with similar outcome; ns, not significant. **p < 0.01 and ****p < 0.0001.

To assess the effect of *C.f/L*-extract in the outcome of *T. congolense* infection, we used Balb/c mice which are reported to be highly susceptible to the infection as they fail to survive the first wave of parasitemia and succumb to the disease within two weeks of infection (Uzonna et al., 1999; Duleu et al., 2004). Balb/c mice were infected with *T. congolense* and treated daily with *C.f/L*-extract at 0.5 μg/g or 2.5 μg/g from days 3 to 8, post-infection. Parasitemia and survival in the *C.f/L*-extract-treated mice were monitored and compared with infected PBS-treated controls. We observed a significant decrease in parasitemia (Figure 2A) and improved survival (Figure 2B) in Balb/c mice treated with 2.5 μg/g of *C.f/L*-extract. However, this was not observed in the group treated with the lower 0.5 μg/g *C.f/L*-extract dose or the PBS treated control group. These findings show that treatment with *C.f/L*-extract decreases parasitemia and promotes host survival following *T. congolense* infection, suggesting that the extract may have trypanocidal properties.

### C.F/L-extract regulates IL-10 and IFN-γ expression in CD4^+^ T cells following *Trypanosoma Congolense* infection in mice

**Figure 3:**
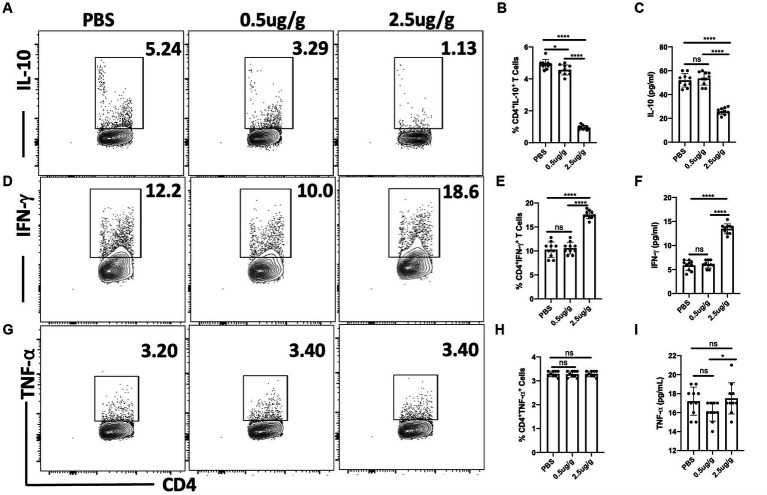
*Crosspteryx fibrifuga* leaf extract (*C.f/L*-extract) regulates IFN-γ and IL-10 but not TNF-α expression in CD4^+^ T cells in *T. congolense*-infected mice. Balb/c mice were infected intraperitoneally (i.p) with 1 × 10^3^ *T. congolense* clone TC13. After 3 days, infected mice were treated with either PBS or different concentrations *C.f/L*-extract once daily for 7 days and then sacrificed to assess to cytokine response in the spleens directly *ex vivo* by flow cytometry. Shown are the frequency of CD4^+^IL-10^+^ **(A,B)**, CD4^+^IFN-γ^+^ **(D,E)** and CD4^+^TNF-α^+^ **(G,H)** T cells in the spleens infected and treated mice. The amount of IL-10 **(C)**, IFN-γ **(F)**, and TNF-α **(I)** in the plasma of infected and treated mice were assessed by ELISA. Results presented are representative of 2 different experiments with similar outcome: ns, not significant. *p < 0.001 and ****p < 0.0001.

It has previously been reported that cytokines including IL-10, IFN-γ, and TNF-α regulate the host response to *T. congolense* infection (Silva et al., 1995; Namangala et al., 2001). We, therefore, investigated whether treatment with *C.f/L*-extract would alter the levels of these cytokines following *T. congolense* infection. Flow cytometry analysis showed decreased frequencies of CD4^+^ IL-10-producing cells in the spleens of Balb/c mice treated with 0.5 μg/g and 2.5 μg/g of *C.f/L*-extract when compared with the control group (Figures 3A,B). In addition, the quantity of secreted IL-10 in the plasma of infected mice were significantly reduced following treatment with 2.5 μg/g of *C.f/L*-extract (Figure 3C). IFN-γ production from CD4^+^ T cells in the spleen as well as plasma levels were only significantly elevated in mice treated with 2.5 μg/g of the extract (Figures 3D–F). We did not observe any significant difference in the frequency of TNF-α^+^CD4^+^ cells following treatment with the extract (Figures 3G,H). However, similar to IFN-γ, plasma levels of TNF-α were significantly increased in mice treated with 2.5 μg/g compared to those treated with 0.5 μg/g or PBS controls (Figure 3I). Taken together, these findings suggest that the administration of *Crosspteryx fibrifuga* leaf extract modulates cytokine production in *T. congolense* infected mice by increasing protective IFN-γ responses and downregulating the suppressive IL-10 responses, an occurrence which is beneficial for *T. congolense* control (Namangala et al., 2001; Onyilagha et al., 2015).

### Pro-inflammatory cytokines and nitric oxide secretion are enhanced in bone marrow derived macrophages (BMDMs) exposed to *C.F/L*-extract

**Figure 4:**
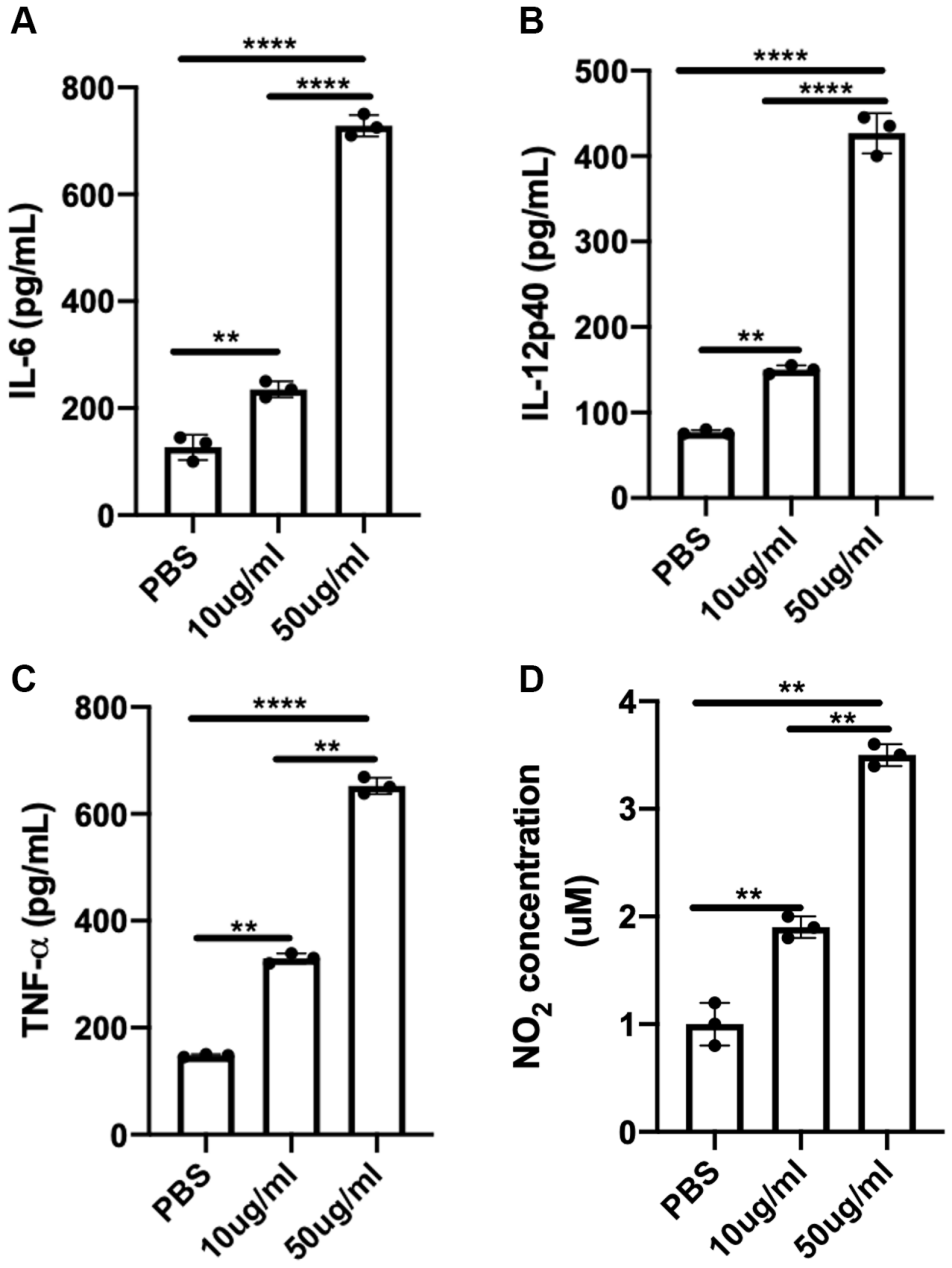
*C.f/L*-extract induces the production of pro-inflammatory cytokines and nitric oxide by bone marrow-derived macrophages. Bone marrow-derived macrophages (BMDMs) from Balb/c mice were treated with either PBS or different doses (10 μg/mL and 50 μg/mL) of *C.f/L*-extract. After 24 h, the supernatant fluids were collected and the amount of IL-6 **(A)**, IL-12p40 **(B)**, and TNF-α **(C)** was determined by ELISA. Griess assay was used to determine the amount of nitric oxide secreted in the culture supernatant fluids. **(D)**. Results presented are representative of 3 independent sets of experiments with similar results. **p < 0.01 and ****p < 0.0001.

An optimal pro-inflammatory or Th1 cytokine response is essential for mounting protective host immune response against *T. congolense* infection (Uzonna et al., 1999; Galvão Da Silva et al., 2003; Holt et al., 2016). Macrophages are critical host cells that contribute to resistance against African trypanosomes, in part by phagocytosis and the production of nitric oxide as well as other inflammatory cytokines (Namangala, 2012). Thus, we assessed the production nitric oxide and pro-inflammatory cytokines by BMDMs after 24 h following stimulation with *C.f/L*-extract. We found that the quantity of IL-6 in the culture supernatant fluids (Figure 4A) was significantly higher in BMDMs treated with the extract (at both 10 μg/mL & 50 μg/mL) when compared to PBS control. Similarly, IL-12p40 (Figure 4B) and TNF-α (Figure 4C) cytokine levels also showed marked elevation in the supernatant fluids, with the effect being more pronounced in BMDMs exposed to the 50 μg/mL extract dose. In addition, *C.f/L*-extract induced increased production of NO in BMDM cultures in a dose dependent manner when compared to PBS control (Figure 4D). These data suggest that *C.f/L*-extract potentially induces a pro-inflammatory immune environment in macrophages, which may contribute to the anti-trypanosomal effects of *C.f/L*-extract in *T, congolense* infected Balb/c mice.

### *C.F/L*-extract is trypanocidal *In vitro*

**Figure 5:**
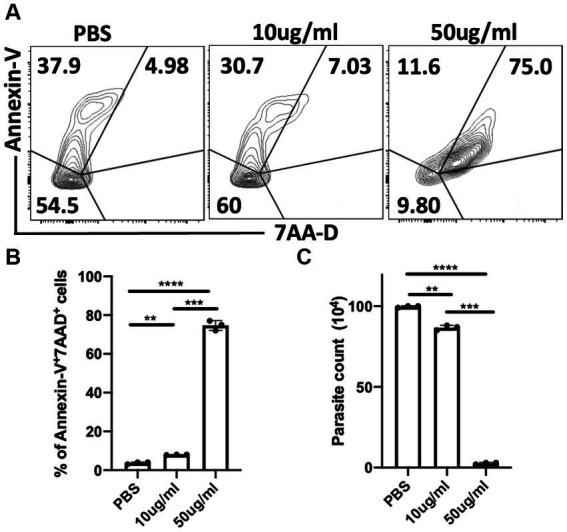
*Crosspteryx fibrifuga* leaf extract (*C.f/L*-extract) is trypanocidal *in vitro* in a dose dependent manner. *Trypanosoma*. *congolense* (1 × 10^6^) parasites were cultured in axenic medium containing Tris-saline glucose (TSG) in the presence or absence of different concentrations of *C.f/L*-extract (10 μg/mL and 50 μg/mL). After 24 h, the viability of the parasites was analysed by Annexin-V and 7AAD staining by flow cytometry **(A)** and the frequency of dead parasites (Annexin-V^+^7AAD^+^) was graphically represented **(B)**. The parasites numbers in the cultures were estimated by counting with a haemocytometer **(C)**. Results presented are representative of 3 different experiments with similar outcome; **p < 0.01, ***p < 0.001, and ****p < 0.0001.

To further understand the potential mechanism by which *C.f/L*-extract treatment decreases parasitaemia in *T. congolense* infected Balb/c mice, we treated *in-vitro* axenic cultures of *T. congolense* with varying doses of *C.f/L*-extract (10 μg/mL & 50 μg/mL) and assessed their viability after 24 h by flow cytometry. We found that the viability of the parasites were compromised in cultures treated with 50 μg/mL of *C.f/L*-extract. This was evidenced by a significant increase in the frequency of Annexin-V^+^7AAD^+^ (dead parasites) (Figures 5A,B). In addition, direct microscopic parasite count using a hemocytometer showed a marked decrease in the numbers of live parasites in *C.f/L* extract-treated wells (Figure 5C), further validating the flow cytometry results. Taken together, these results suggest that *C.f/L*-extract may control parasite burden in infected mice by directly killing *T. congolense* parasites.

### *Crosspteryx fibrifuga* leaf (*C.F/L*-extract) disrupts superoxide dismutase activity in *Trypanosoma Congolense* parasites

**Figure 6:**
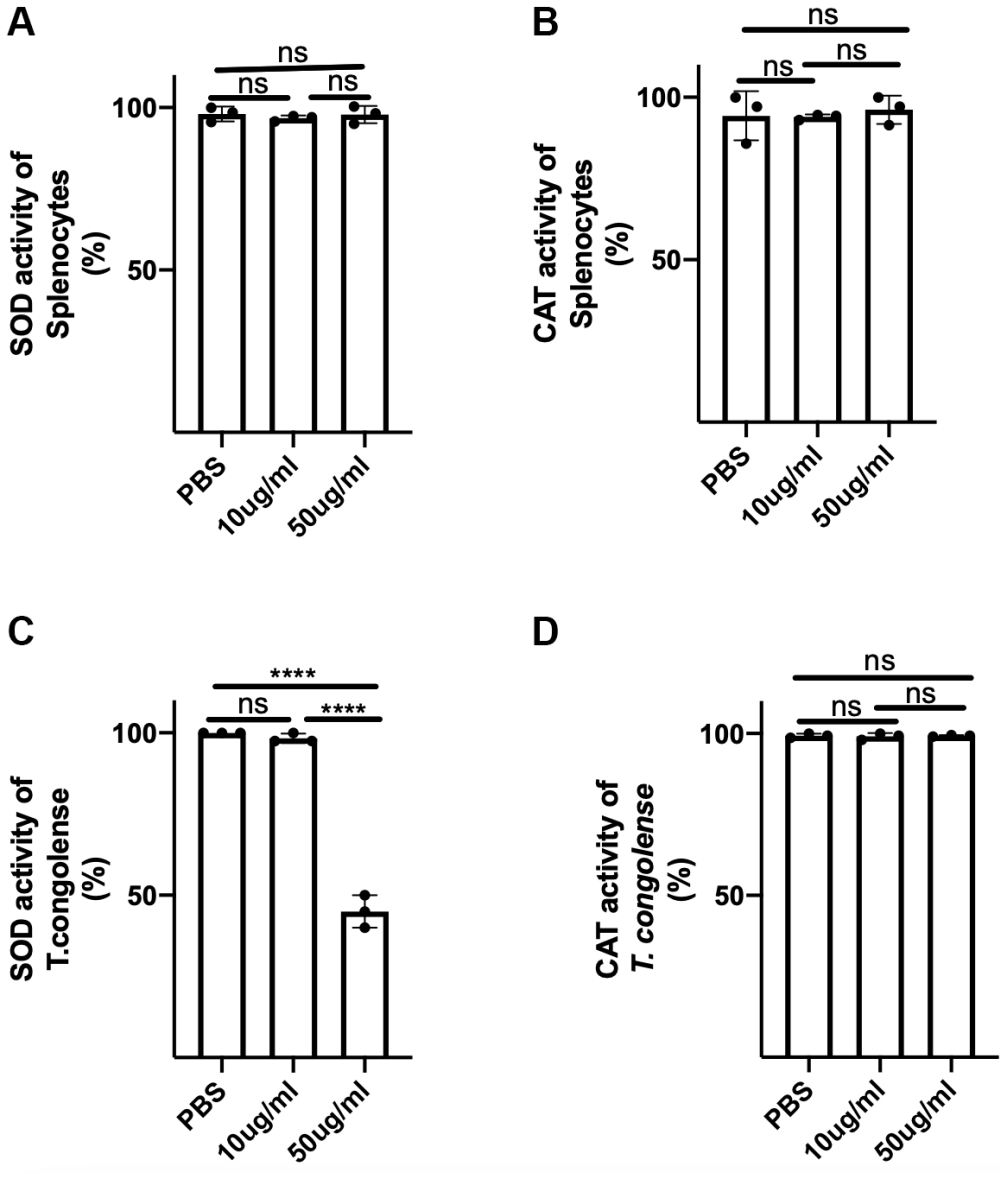
*C.f/L*-extract selectively disrupts superoxide activity in *T. congolense* parasites. Splenocytes (5 × 10^6^) or freshly isolated *T. congolense* parasites (20 × 10^6^) were cultured in complete RPMI culture medium or Tris-saline glucose (TSG), respectively, in the presence or absence of *C.f/L-extract* (as indicated). After 24 h, the cells and parasites were pelleted and homogenized or sonicated (parasites) and the supernatants from the homogenized cell pellets were assayed for superoxide dismutase (SOD), **(A,D)** and catalase (CAT), **(B,D)** activity using specialized kits. Results are pooled from 3 different experiments with similar outcome; ns, not significant. ****p < 0.0001.

Next, we investigated the potential mechanism of direct parasite death following exposure to *C.f/L*-extract. Since it has been reported that superoxide anions and free radicals are trypanocidal (Martínez et al., 2019) and that trypanosomes use superoxide dismutase (SOD) and catalase (CAT) to counteract the trypanocidal efficacy of free radicals and superoxide anions (Freire et al., 2017; Martínez et al., 2019), we examined whether *C.f/L*-extract disrupted the activity of these parasite-shielding enzymes by assessing the activities of these enzymes in supernatant fluids obtained from pelleted and sonicated parasites treated with *C.f/L*-extract. We found SOD activity was markedly reduced in parasites exposed to 50 μg/mL of C.f/L-extract (Figures 6A–D). Interestingly, we did not observe any difference in the activity of catalase in *T. congolense* parasites following treatment with *C.f/L*-extract (Figure 6D). Taken together, these findings suggest that *Crosspteryx fibrifuga* leaf extract mediated its trypanocidal effect in a concentration dependent manner, by selectively disrupting the parasite-shielding molecule, superoxide dismutase enzyme activity in *T. congolense* parasites.

## Discussion

Although there have been advances in the treatment of *Trypanosoma* infections over the years, effective elimination of the disease in endemic areas remains a major challenge. The absence of an effective vaccine for prophylaxis, and the issue of drug resistance and associated toxicity, support the need to develop better alternative treatment. In this study, we assessed the anti-trypanosomal effect of *Crosspteryx fibrifuga* leaf (*C.f/L*-extract) *in vitro* and *in vivo* in a mouse model of experimentally induced African trypanosomiasis (*T. congolense*). Our results show that *C.f/L*-extract is trypanocidal as evidenced by marked decrease in parasite burden in *in-vitro* cultures as well as in infected animals. We further showed that this trypanocidal effect was associated with the disruption of superoxide dismutase (SOD) activity. This disruption of SOD activity in the parasites may enhance their susceptibility to reactive superoxide free radicals since it has been shown that trypanosomes use this enzyme to counteract the trypanocidal efficacy of free radicals and superoxide anions (Martínez et al., 2019). The use of SOD by micro-organisms as an evasive mechanism against host cells has previously been reported in other parasitic diseases and is thought to be one of the major evasion mechanisms favouring *Leishmania* survival in macrophages (Ghosh et al., 2003). SOD has also been implicated as an evasion mechanism in *Plasmodium falciparum* (Dive et al., 2003), *Trypanosoma brucei* (Prathalingham et al., 2007), and *Haemonchus contortus*, a pathogenic nematode of veterinary importance (Rashid and Irshadullah, 2014).

In host infections with protozoan parasites such as trypanosomes and *Leishmania*, a protective host response is often characterized by the timely release and balance of specific immune mediators like the pro-inflammatory Th1 cytokine, IFN-γ, from T cells (Kumar and Tarleton, 2001; Ikeogu et al., 2021). Alternatively, regulatory T cells secrete IL-10, the cytokine tasked with suppressing these pro-inflammatory responses (Hertz et al., 1998; Namangala et al., 2001). Additionally, TNF-α, another cytokine also produced by immune cells such as macrophages, natural killer (NK) cells and T cells, are important for resistance to infection (Silva et al., 1995). We found that treatment of *T. congolense* infected mice with *C.f/L*-extract led to the elevated secretion of IFN-γ at the expense of IL-10 from T cells, thereby highlighting the immune modulating potential of this extract following *T. congolense* infection. The lack of *C.f/L*-extract effect on TNF-α release from T cells *in vivo*, suggests a CD4^+^ T cell-TNF-α independent mechanism in its anti-trypanosomal action *in vivo*. Furthermore, treatment of BMDMs with *C.f/L*-extract *in vitro* showed an upregulation of pro-inflammatory cytokines, such as IL-6, IL-12p40, and TNF-α. This observation, suggests a possible contribution of macrophages (via TNF-α) to the *C.f/L*-extract mediated anti-trypanosomal response *in vivo* (Silva et al., 1995). On the other hand, anti-trypanosomal effect of *C.f/L*-extract in infected mice could be indirect via IL-6 (Sanmarco et al., 2017) and IL-12p40 (Galvão da Silva and de Almeida, 2001; Galvão Da Silva et al., 2003; Graefe et al., 2003) by mediating the differentiation of naïve CD4^+^ T cells to CD4^+^ IFN-γ^+^ Th1 cells that have been shown to contribute to immunity to experimental African trypanosomiasis (Galvão da Silva and de Almeida, 2001; Galvão Da Silva et al., 2003; Graefe et al., 2003). Additionally, we observed a concentration dependent increase in nitric oxide (NO) production from macrophages following treatment with *C.f/L*-extract. Nitric oxide is thought to be beneficial to the host against a wide species of pathogens including trypanosomes (Vespa et al., 1994; Silva et al., 1995). Often secreted by macrophages, NO directly acts on the parasite, causing oxidative damage and thereby inhibit their growth and multiplication (Vespa et al., 1994; Silva et al., 1995).

Yusuf et al. (2006) observed that the treatment of rats with aqueous stem bark extract from *C. febrifuga* had no anti-trypanosomal activity (Yusuf et al., 2006), which is in contrast to our observations. Aside the fact that the stem bark of the *C. febrifuga* plant was used in their study (which is different from the leaf extract used in this study), their route of administration (oral) was also different from the intraperitoneal route employed in our study. The oral route of administration exposes the extract to breakdown by digestive enzymes (Gavhane and Yadav, 2012); thus only sub-optimal concentrations of extract were able to potentially reach the blood (Gavhane and Yadav, 2012). The difference in the specie/breed of the host used in their study, may also play a role in the different results observed in this present study, as rats may have different pharmacodynamics response to test agents (Pan et al., 2016; Tyson et al., 2020). Also, unlike Yusuf et al. (that used aqueous extract), our study utilized a methanolic extract of *C.f/L*. Methanol has been shown to be efficient at extracting organic compounds compared to water (Truong et al., 2019). In addition, high-performance liquid chromatography (HPLC) of the extract would provide further insights to the phytochemical constituents of the leaves or stem bark extracts of *C. febrifuga*. This may help elucidate the actual constituent(s) responsible for the anti-trypanosomal effect observed in our study.

An important observation in our report is the selective toxicity of *C.f/L*-extract against the parasites (without compromising the host cells). The capacity of a drug to selectively target microbial agents with little or no damage to the host cells and tissues is one of the hallmarks of an ideal anti-microbial agent (Purssell, 2019), and *C.f/L*-extract shows promise in this regard at lower concentrations. While our study does not fully investigate or elucidate the exact phytochemical constituents of *C.f/L*-extract, it has been reported that the leaf extracts contain sufficient quantity of the flavonoid quercetin (Crossopteryx febrifuga, 2021), which is a pro-oxidant molecule that can disrupt parasite mitochondrial function and also promote the generation of reactive oxygen species (Fonseca-Silva et al., 2011).

Superoxide dismutase (SOD) has been shown to be anti-apoptotic and rescues cells from apoptosis. Greenlund et al. (1995) showed that apoptosis induced in neuronal cells following nerve growth factor starvation was significantly delayed when those nerve cells were treated with SOD or transfected with SOD cDNA (Greenlund et al., 1995). We did not observe a disruption in SOD activity in host cells possibly because the cells were grown in a stress-free environment (using complete media). In addition, we also did not observe marked cell death in host cells exposed to *C.f/L*-extract. The protective effect of SOD in trypanosomes was previously demonstrated by Radhika et al who showed that genetic ablation of SOD gene (*sodb1*) in *T. brucei* parasites increased the sensitivity of the mutant parasites to free radicals induced by drugs such as Nifurtimox and Benznidazole (Prathalingham et al., 2007). Furthermore, in an acute model of American trypanosomiasis (Chaga’s disease), Francisco et al showed that the inhibition of SOD activity resulted in decreased parasitemia in *T. cruzi* infected Balb/c mice (Olmo et al., 2015). These findings show the critical contribution of SOD to parasite survival in oxidative environments. We also report a similar observation, as we showed that *Crossopteryx febrifuga* leaf extract led to significant disruption in SOD activity *T. congolense* parasites, leading to their death *in vitro* and a corresponding decrease in parasitemia *in vivo*.

In summary, we have shown that *Crossopteryx febrifuga* leaf extract modulates the host immune response to *Trypanosoma congolense* infection. This plant has been adopted in traditional medicine in parts of western Africa as anti-malarial, anti-pyretic and anti-protozoan agent (Adeola et al., 2011; Salawu et al., 2013). In our study, treatment of mice infected with *T. congolense* with *C.f/L*-extract led to reduction in parasitemia and an associated increased survival period. Additionally, there was increased production of pro-inflammatory cytokines and other immune mediators that boost the host immune response against the parasite. *C.f/L*-extract also showed a targeted toxicity against the parasite without compromising host cells, by selectively disrupting the parasite defense mechanism against oxidative stress (decreased SOD activity). This is the first report showing the potential beneficial use of *C.f/L*-extract in the treatment of experimental African trypanosomiasis caused by *T. congolense* infection in mice. These findings show that *Crossopteryx febrifuga* leaf extract is a potential therapeutic agent for the treatment of African trypanosomiasis.

## Data availability statement

The original contributions presented in the study are included in the article/supplementary material, further inquiries can be directed to the corresponding authors.

## Ethics statement

The animal study was approved by University of Manitoba Animal Care Committee. The study was conducted in accordance with the local legislation and institutional requirements.

## Author contributions

NI: Data curation, Formal analysis, Investigation, Methodology, Writing – original draft, Conceptualization, Writing – review & editing. FO-A: Data curation, Formal analysis, Investigation, Methodology, Writing – original draft, Writing – review & editing. CE: Writing – review & editing, Formal analysis, Investigation, Methodology. PJ: Writing – review & editing, Investigation, Methodology. AM: Writing – review & editing. JO: Writing – review & editing, Conceptualization, Project administration, Resources, Validation. JU: Conceptualization, Writing – review & editing, Funding acquisition, Supervision. CO: Writing – review & editing, Formal analysis.
